# Spray-Dried Amorphous Solid Dispersions of Atorvastatin Calcium for Improved Supersaturation and Oral Bioavailability

**DOI:** 10.3390/pharmaceutics11090461

**Published:** 2019-09-06

**Authors:** Jaewook Kwon, Bhupendra Raj Giri, Eon Soo Song, Jinju Bae, Junseong Lee, Dong Wuk Kim

**Affiliations:** College of Pharmacy & Research Institute of Pharmaceutical Sciences, Kyungpook National University, Daegu 41566, Korea

**Keywords:** atorvastatin calcium, hydroxypropyl methylcellulose (HPMC), amorphous solid dispersion, spray-drying, solubility enhancement, bioavailability

## Abstract

Over the past few decades, the amorphous solid dispersions (ASDs) technique has emerged as a promising strategy to enhance the in vitro/in vivo characteristic of hydrophobic drugs. The low aqueous solubility and poor bioavailability of atorvastatin calcium (ATO), a lipid-lowering drug, present challenges for effective drug delivery. The objective of this work was to improve the aqueous solubility, in vitro dissolution, and oral absorption of ATO with amorphous solid dispersion technique prepared by spray-drying method. The optimized ternary formulation comprising of ATO; hydroxypropyl methylcellulose (HPMC), as a hydrophilic polymer; and sodium lauryl sulfate (SLS), as a surfactant, at a weight ratio of 1/1/0.1, showed significant improvement in aqueous solubility by ~18-fold compared to that of the free drug, and a cumulative release of 94.09% compared to a release of 59.32% of the free drug. Further, physicochemical studies via scanning electron microscopy, differential scanning calorimetry, and powder X-ray diffraction revealed a change from the crystalline state of the free drug to its amorphous state in the ASD. Pharmacokinetic analysis in rats demonstrated 1.68- and 2.39-fold increments in AUC and *C*_max_, respectively, in the ASD over the free drug. Altogether, hydrophilic carrier-based ASDs prepared by the spray-drying technique represent a promising strategy to improve the biopharmaceutical performance of poorly soluble drugs.

## 1. Introduction

Approximately 40% of new chemical entities identified in drug discovery programs have poor aqueous solubility [1,2]. Although these chemical entities possess potentially desirable pharmacodynamic properties, they fail to reach the market due to their low bioavailability. Over the past few decades, several methods have been employed to improve drug solubility, including salt formation [3], use of prodrug strategies [4], particle size reduction (micronization and nanonization) [5], complexation [6], use of lipid based formulations [7], and use of solid dispersions [8,9,10].

Solid dispersion (SD), a concept introduced by Sekiguchi and Obi in the 1960s [11,12], is the dispersion of a hydrophobic drug in one or more hydrophilic carrier (matrix), where the drug can be present in a solubilized, fine crystalline, or amorphous state [13]. Today, SD has become one of the most widely accepted and promising approaches for improving drug solubility leading to enhanced bioavailability [14]. Increase in surface area via particle size reduction, molecular dispersion of crystalline compounds into hydrophilic carriers resulting in improved wettability and enhanced porosity, and manipulation of the solid state of the drug substance, i.e., the transformation of a crystalline drug to its amorphous state, all of which result from use of SD systems, leading to improved in vitro and in vivo drug characteristics [15]. Common methods used to prepare ASDs include quench cooling from the melt, rapid solvent evaporation, melt extrusion, spray-drying, and electrospinning/spraying. Spray-drying is a commonly used solvent evaporation technique for the production of SDs and is extensively used in pharmaceutical industry. It mainly involves dissolving or suspending or drug and carrier(s), atomization of the solution/suspension, mixing and drying of liquid with a stream of heated air, and separation of the dried particles from the hot gas [16].

The carriers used for drug encapsulation have a major influence on the release profile of the dispersed drug. Such carriers include hydroxypropyl cellulose, hydroxypropyl methylcellulose (HPMC), ethyl cellulose, cellulose acetate phthalate, ethyl acetate, chitosan, and methacrylic acid copolymers [17]. HPMC is an attractive nonionic, water-soluble, cellulose ether derivative. It is a semisynthetic and viscoelastic cellulose molecule associated with ease of manufacturing of oral solid dosage forms (OSDFs), and is accepted globally by regulatory bodies [18].

**Figure 1:**
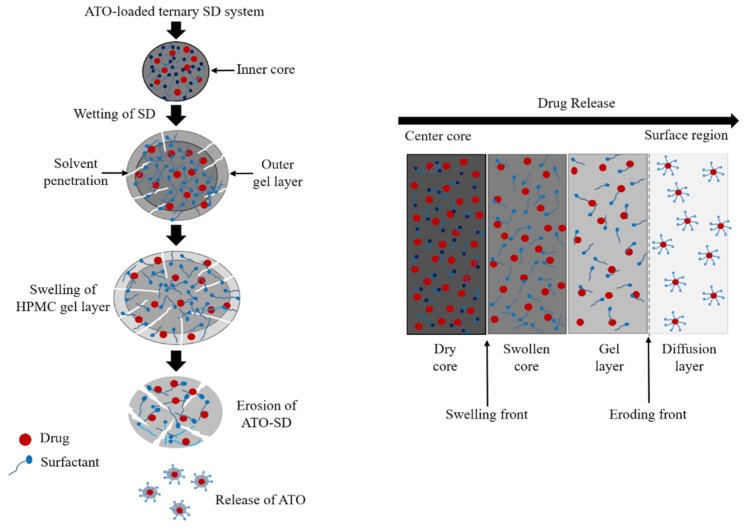
General schematic diagram showing drug release from a ternary solid dispersion with a hydroxypropyl methylcellulose (HPMC) carrier and a surfactant. Initial wetting hydrates the polymers and forms a gel layer around the SD. Penetration of water leads to expansion of the gel layer. Finally, as the water penetrates further into the core, the outer carrier layer becomes completely hydrated and dissolves, releasing the encapsulated drug.

The mechanism of drug release from solid dispersions containing HPMC matrix involves a complex phenomenon [19], typically involving wetting, liquid penetration into the dry core matrix, polymer gelatinization (polymer dissolution in a semidilute state), polymer dissolution, and diffusion/erosion of the solubilized drug through the gel layer. Concomitantly, outer layers of the SD system become fully hydrated and dissolve, referred to as erosion, eventually releasing the encapsulated drug. As the outer layer undergoes complete hydration and dissolution, the inner layers begin to continuously replace it [20] (Figure 1). Generally it is presumed that the hydrophilic drugs are released mainly through diffusion from the HPMC gel layer whereas, the hydrophobic drugs are largely released via erosion of the gel layer [21]. These processes are mainly affected by interactions of the drug with the hydrophilic polymer used for incorporation and formation of a drug-loaded carrier matrix.

A third-generation SD system is developed with the addition of a third agent or more excipients along with a polymer carrier (so called ternary SD), in order to prevent drug recrystallization during storage. These third agents commonly include surfactants such as, polyethylene-polypropylene glycol, bile salt, poloxamers, polysorbates, sodium lauryl sulfate (SLS), alkyl benzene sulfonates, benzalkonium chloride, etc., which can improve the solubility and dissolution process and stabilize the amorphous form of drug in SD. Further, the surfactants can behave as a precipitation inhibitor to further increase the degree of supersaturation in vivo [22,23]. Therefore, careful polymer and surfactant selection is crucial in gaining the maximum advantage of ASDs and avoiding failure through drug recrystallization.

Atorvastatin calcium is a synthetic lipid-lowering drug, generally prescribed in the treatment atherosclerosis and coronary disease with or without other lipid lowering agents [24,25]. ATO is the most preferred drug candidate among other statins that are indicated for treatment of moderate to severe familial or non-familial hypercholesterolemia [26,27]. LIPITOR^®^ tablets (brand of atorvastatin calcium) are commercially available in different strengths, including 10, 20, 40 or 80 mg. ATO as a drug candidate was selected because of its low oral bioavailability (12% from a 40 mg oral dosage form), poor aqueous solubility, crystalline nature, and high hepatic first-pass metabolism [28,29]. Thus, to gain maximum pharmacotherapeutic actions, the aqueous solubility of ATO needs to be improved. We hypothesized that the in vitro and in vivo characteristics of atorvastatin calcium (ATO) would be improved by incorporating the drug into a cellulose matrix. Several studies have been carried out with ATO, such as preparation of ATO SDs [30,31,32], size reduction (i.e., ATO nanopreparations) [33]. However, the influence of the cellulose matrix system on the preparation and characterization ATO-loaded ternary solid dispersion system is an area of interest that needs investigating. Therefore, the overall objective of the present study is to investigate the influence of a cellulose polymer in an atorvastatin calcium (ATO)-loaded ternary amorphous solid dispersion system with the goal of improving aqueous solubility and dissolution profile, and thus enhancing the oral bioavailability of the drug. The ATO-ASDs comprising the drug, HPMC, and sodium lauryl sulfate (SLS) were prepared via a spray-drying technique. The solid dispersions were characterized by scanning electron microscopy (SEM), differential scanning calorimetry (DSC), and X-ray powder diffraction (XRD). The dissolution profiles of the formulations were assessed in vitro, while the oral bioavailability of ASD was quantified in rats.

## 2. Materials and Methods

### 2.1. Materials

ATO was kindly provided by Hanmi Pharm. Co. (Suwon, Korea). Hydroxypropyl methylcellulose (HPMC, 2910) and sodium lauryl sulfate (SLS) were purchased from Shin-Etsu Co. (Tokyo, Japan) and Duksan Chemical Co. (Ansan, Korea), respectively. All other chemicals and reagents were of HPLC-grade and were used without further purification.

### 2.2. Screening of Excipients

For the selection of a suitable solid matrix former, various hydrophilic polymers and surfactants were screened using solubility testing published guidelines with slight modifications [34]. An excess of drug was added to 1 mL of distilled water containing 1% (*w*/*v*) polymer or surfactant and vortexed for a few seconds. The microtubes were shaken at 25 °C and 100 rpm for five days in an isothermal water bath shaker. The resulting suspensions were centrifuged at 10,000 g for 10 min and the supernatant was filtered through a 0.45 µm membrane filter. The filtrate was diluted with methanol as needed and then assayed using an Agilent 1260 Infinity LC system (Agilent Technologies; Santa Clara, CA, USA) equipped with Agilent ChemStation software (version B.04.02), an Agilent 1260 Quat pump, and an Capcell Pak C18 column (Shiseido, 250 mm × 4.6 mm I.D., 5 μm). The mobile phase consisted of methanol, acetonitrile, and distilled water (*v*/*v*/*v*: 40/40/20), at a flow rate of 1 mL/min. The mobile phase was monitored using an Agilent 1260 VWD detector (Agilent Technologies; Santa Clara, CA, USA) at a wavelength of 240 nm, and the injection volume was 10 μL. All the experiments were carried out in triplicate (*n* = 3), and the solubility data are represented as mean ± standard deviation.

### 2.3. Preparation and Optimization of ATO-Loaded Solid Dispersions

The polymer and surfactant demonstrating the highest aqueous drug solubility in screening tests were selected for investigating the optimal SD formulation. A Büchi B-290 nozzle-type mini spray dryer (Buchi Co.; Flawil, Switzerland) was employed for the preparation of ATO-loaded solid dispersions. Various ratios of HPMC and SLS (fixed at 0.1 g) were dissolved in 300 mL of water. Then, 150 mL of ethanol containing 1 g of drug was added to the aqueous solution (Table 1). Each resulting solution was continuously stirred and transferred to a 0.7 mm pneumatic nozzle using a peristaltic pump and spray-dried, leading to production of ATO-loaded solid dispersions. The spray-drying conditions were inlet and outlet temperature of 90 and 69–71 °C, respectively; a feed rate of 10 mL/min; and 100% aspiration. A total of five SDs were prepared, and aqueous solubility testing of the drug in SDs was carried out to select the optimized formulation.

### 2.4. Solubility of ATO in Solid Dispersions

An excess amount of ATO-loaded SDs (equivalent to about approximately 100 mg of free ATO) was added to distilled water, vortexed, and agitated at 25 °C for five days. The resulting suspensions were centrifuged at 10,000 g for 10 min (Hanil; smart 15 plus), and the supernatant was filtered through a 0.45 μm membrane filter and diluted with methanol. The concentration of ATO in the samples was assayed using HPLC as described above.

### 2.5. In Vitro Dissolution Study

The in vitro drug release of free drug, physical mixture, and optimized ATO-loaded SDs was carried out in a USP type II dissolution apparatus. The SD samples equivalent to 20 mg of ATO were filled into hard gelatin capsules (size 0) and subjected to dissolution testing (ERWEKA; DT 620, Heusenstamm, Germany) with 900 mL of distilled water as the medium, maintained at a constant temperature (37 ± 0.5 °C) with a paddle rotation speed of 100 rpm. In several publications involving ATO, water was used as a dissolution medium in the in vitro dissolution study [31,32], and therefore water was chosen as an in vitro dissolution medium in this work. At a predetermined time intervals (5, 10, 15, 30, 45, 60, and 90 min), 2 mL of the medium was sampled and an identical amount of fresh dissolution medium was immediately replenished to compensate for the loss during sampling. Prior to analysis, the samples were filtered through a 0.45 μm membrane filter. A 10 µL aliquot of the sample was injected into the HPLC system and the concentration quantified using the method described above.

### 2.6. Physicochemical Characterization

#### 2.6.1. Loading Efficacy

The SDs equivalent to 10 mg ATO were completely dissolved in 100 mL methanol, filtered through 0.45 μm membrane filter, and assayed for the content of drug by HPLC as mentioned above. The loading efficacy (%) was calculated as follows; loading efficacy (%) = *C*_a_/*C*_t_ × 100, where *C*_a_ and *C*_t_ were the actual and theoretical drug content in these SDs, respectively.

#### 2.6.2. Scanning Electron Microscopy (SEM)

The shape and surface morphology of the drug, carrier, surfactant, and SD formulation were examined using SEM (SU8220; Hitachi; Tokyo, Japan) operating at an accelerated voltage of 5.0 kV. Samples were affixed onto a brass specimen holder using double-sided adhesive tape, and the powders were made electrically conductive by coating with platinum (6 nm/min) in vacuum (0.8 Pa) using an EmiTeck Sputter Coater K575 K (Quorum Technologies Ltd.; West Sussex, UK) for 4 min at 15 mA.

#### 2.6.3. Differential Scanning Calorimetry (DSC)

The crystallinity of free ATO, carrier, surfactant, and SD were further investigated using a TA DSC Q20 instrument (TA Instruments; Newcastle, DE, USA). Approximately 5 mg of sample was weighed, sealed, and placed in an aluminum pan. The samples were heated from 30 °C to 220 °C at a constant temperature change rate of 10 °C/min under nitrogen gas flow at 50 mL/min. A physical mixture (PM) was prepared by mixing the polymer, surfactant, and the drug at the same weight ratio as that of the optimized ATO-loaded SD (F2).

#### 2.6.4. X-ray Powder Diffraction

X-ray powder diffraction (XRD) evaluation of the samples was done using a D/MAX-2500 XRD instrument (Rigaku, Japan) equipped with a copper anode operated using Cu K_α_ radiation (1.54178 Å, 40 kV, and 40 mA). The samples were scanned from 5° to 35° using step scan mode with a step size of 0.05°/s at room temperature and a 2θ diffraction angle to obtain the diffraction patterns.

### 2.7. Pharmacokinetic Studies

All animal care and procedures were performed in accordance with the guidelines and protocols approved by the Institutional Animal Care and Use Committee (IACUC) at Kyungpook National University (Permit Number: 2018-0129, Date of approval: 01 September 2018). The bioavailability of ATO in the SD was evaluated in 7–9-week-old, male Sprague Dawley rats, weighing 250–280 g (Samtako Bio Korea, Osan, Korea). The animals were randomly divided into two groups of six rats each. These rats were kept at controlled conditions of 25 ± 2 °C/55 ± 5% RH and fasted for 12 h prior to experiments with free access to water.

The rats were individually administered a 0.75 mL aqueous suspension of free ATO or SD (F2) equivalent to 30 mg/kg of ATO via oral gavage. Approximately 0.35 mL of blood was sampled via the jugular vein at predetermined time intervals and centrifuged at 8000 g for 10 min at 4 °C to separate the plasma. The plasma samples were stored at −20 °C until further studies. To 90 µL of each plasma sample, 100 µL of acetonitrile and 10 µL of internal standard (ibuprofen in methanol, 100 µg/mL) was added and vortexed for a few seconds. Then, the samples were centrifuged at 13,000 g for 10 min at 4 °C and the supernatants immediately transferred to vials for HPLC quantification. The optimized HPLC conditions [35] were used in this study. Briefly, a mobile phase composed of methanol, acetonitrile, and potassium dihydrogen phosphate (*v*/*v*/*v*: 20/50/30) adjusted to pH 3.5 with orthophosphoric acid was used and the injection volume was 20 μL. The other HPLC conditions were the same as described in Section 2.2. All pharmacokinetic parameters, including area under the plasma concentration–time curve (AUC), maximum plasma concentration (*C*_max_), time to reach the maximum plasma concentration (*T*_max_), half-life (*t*_1/2_), and elimination rate constant (*K*_el_) were estimated using WinNonlin^TM^ (Pharsight Corp.; Mountain view, CA, USA). Values are reported as mean ± S.D. and the data was statistically significant at *p* < 0.05 between the two formulations checked by the Student’s *t* test.

## 3. Results and Discussion

### 3.1. Selection of Excipients

**Figure 2:**
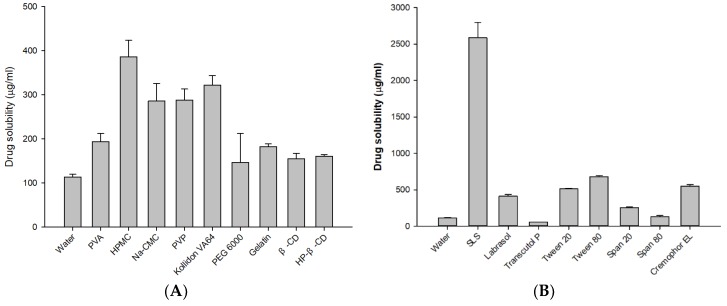
Solubility of atorvastatin calcium (ATO) in a different (**A**) polymer and (**B**) surfactant solutions (1% *w*/*v*) (*n* = 3).

Carrier selection plays a significant role in the performance of amorphous SDs, because the molecular dispersion of drug in the carrier must involve significant miscibility, with strong drug–carrier interactions (e.g., hydrogen-bonding) for stability against recrystallization [36,37]. To derive the maximal advantage from an ASD system, the carrier’s miscibility with the drug should be investigated prior to formulation. The aqueous solubility of ATO in various carriers and surfactants is presented in Figure 2A. Among the carriers tested, HPMC showed the highest drug solubility at 385.82 ± 37.78 µg/mL, whereas PEG 6000 resulted in the lowest at 146.79 ± 65.79 µg/mL. Among the various surfactants tested, drug solubility in SLS was substantially high (2587.92 ± 210.01 µg/mL) (Figure 2B). Therefore, HPMC and SLS were chosen as the hydrophilic carrier and surfactant for the formulation of ATO-loaded ASDs.

HPMC is nontoxic, chemically stable, and inert, and is therefore compatible with numerous drugs; further, it does not show pH-dependent solubility, and is a “generally regarded as safe” molecule, allowing its use in pharmacological applications [38,39]. The free energy of amorphous forms has excess enthalpy and entropy, which makes amorphous forms prone to recrystallization [40]. The addition of a surfactant was found to overcome the limitations associated with the formulation of a solid dispersion system such as, the poor miscibility of drug and polymer, recrystallization of drug in SD system, etc. [22]. Thus, the use of suitable matrix former is required to avoid the formation of stable drug crystals in a solid dispersion. Therefore, we used a low concentration of SLS (0.1 g) as a surfactant along with the HPMC polymer in the formulated ternary ASDs system for enhancement of drug-polymer miscibility and stabilization of the amorphous state drug (prevent recrystallization of ATO) presented in the solid dispersions.

### 3.2. Optimization of ATO-Loaded SD

**Figure 3:**
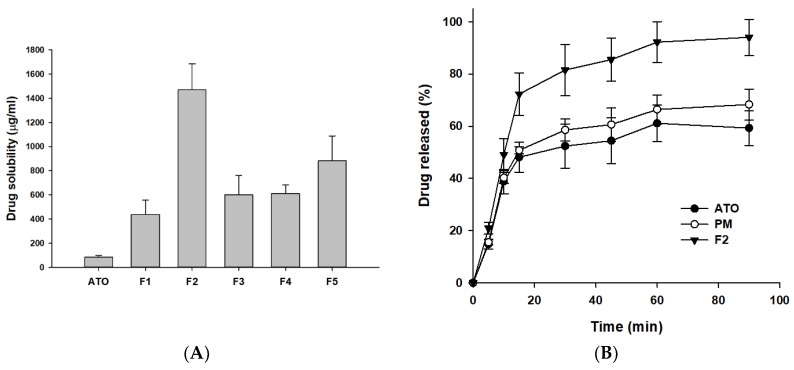
Solubility of ATO in various solid dispersions (SDs) (**A**) and dissolution profile of free ATO, PM, and F2 (**B**). The PM and F2 formulation was composed of ATO/HPMC/SLS at a weight ratio of 1:1:0.1. Each value represents the mean ± S.D (*n* = 3).

The novel ATO-loaded ASD formulations were prepared using a lab-scale spray dryer with water and ethanol, a hydrophilic carrier (HPMC), and a surfactant (SLS). The prepared ATO-loaded ASDs were subjected to solubility studies to investigate the effect of the carriers on the drug solubility. The results in Figure 3A show that irrespective of the ratios of HPMC and SLS, a significant improvement (5.19–17.44-fold) in aqueous solubility of ATO was observed in all the formulations (F1–F5) compared to free drug alone. However, it was found that aqueous drug solubility did not improve with increasing HPMC concentration in the ASD formulations (F3–F5). This could be due to the highest level of supersaturation achieved with F2 formulation (Figure 3A). Once the maximum solubility is attained with F2, the further addition of polymer does not guarantee improved drug solubility which was seen with F3, F4, and F5 formulations. Similar results have been reported for atorvastatin [32], raloxifene [41], valsartan [42], etc. This suggested that choosing an optimal drug-carrier ratio is prerequisite to derive the maximal advantage from use of the ASD system. The highest drug solubilizing effect (i.e., supersaturation) was observed when the drug-carrier ratio was 1:1 (F2 formulation). Therefore, F2 was chosen as the optimized ATO-loaded ASD formulation for further studies.

### 3.3. Evaluation of ATO-Loaded SD

**Figure 4:**
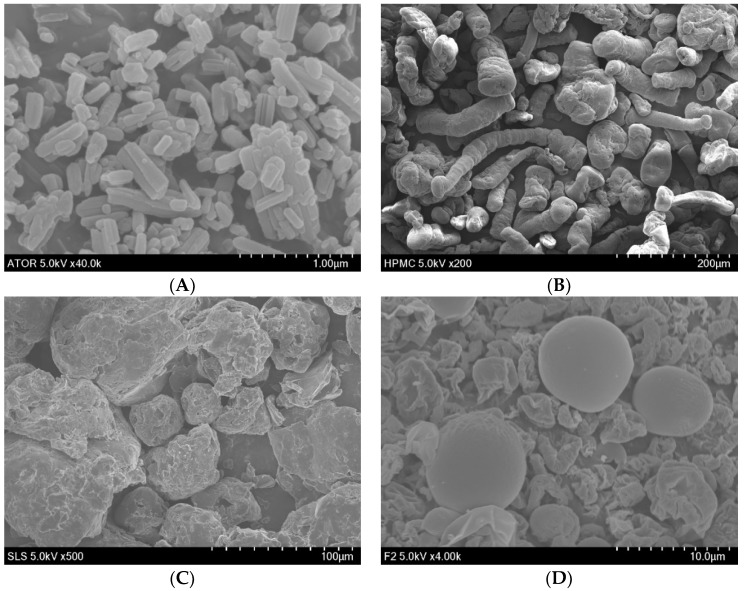
SEM images of (**A**) free ATO (40,000 X), (**B**) HPMC (200 X), (**C**) SLS (500 X), and (**D**) F2 (4000 X).

The dissolution profile of free drug, physical mixture (PM), and the optimized ASD formulation (F2) was assessed using distilled water as dissolution medium. The dissolution profile of F2 showed an initial increase, with approximately 70% drug release within the first 15 min, most probably due to an initial burst release of the hydrophobic drug from the hydrophilic matrix in ASD. One possible explanation is that the dissolution of F2 was accelerated due to the hydrophilic nature of HPMC, leading to rapid water transport into the SD matrix, which resulted in hydration, wetting, and drug release with much faster kinetics from the SD system. In addition, the presence of surfactant (SLS) in the ternary SD improved the drug–polymer miscibility and reduces the interfacial tension between the spray dried particles and the release media. Further, the increased in surface area available for dissolution due to the reduced particle size (observed in SEM image of F2 (Figure 4D)) of SDs would also have assisted in the initial burst drug release from the F2 formulation. Hence, the preparation of ATO-loaded SD essentially showed the initial burst release taking advantage of the increased surface area, amorphous state, and effective wettability of carriers (HPMC and SLS). Similar drug kinetic results have been reported for atorvastatin [30], atorvastatin calcium/ezetimibe [24], itraconazole [43], etc. By 60 min, both the ATO and F2 had dissolved to their maximum state, showing plateaus thereafter. Moreover, F2 showed almost complete, i.e., 90% dissolution within an hour, whereas free ATO showed approximately 60% dissolution within the same time frame (Figure 3B). In addition, PM showed improved drug dissolution than free drug yet lower than that of F2 formulation. The improved dissolution of ATO in physical mixture might be due to the presence of hydrophilic carriers (HPMC and SLS). Thus, the further increased in vitro dissolution of ATO-loaded SD (F2) compared to the bulk drug, as well as the PM, highlighted the significance of the solid dispersion system as a potential technique to enhance the biopharmaceutical characteristics of poorly water-soluble drugs. Overall, the SD formulation of ATO showed a significant increase in dissolution compared to the PM and free drug alone. This increase in dissolution can be explained by the enhanced supersaturation resulting from the molecular dispersion of the poorly water-soluble ATO in the hydrophilic carrier matrix.

### 3.4. Physicochemical Characterization

Physicochemical characterization is useful to examine the solid state properties of drug, carriers and spray-dried solid dispersions. The ATO-loaded SDs prepared in this study gave almost 100% loading efficiency. Scanning electron microscopy revealed the morphological characteristics of free ATO, HPMC, SLS, and F2 (Figure 4). The SEM image of free ATO showed slightly smooth-surfaced, long, cylindrical-shaped articles (Figure 4A), whereas that of F2 showed spherical shaped, smooth-surfaced solid dispersions (Figure 4D). In addition, the SEM images of HPMC and SLS (Figure 4B,C) featured larger rough and irregular-shaped particles. The presence of crystallinity could be anticipated by the appearance of drug particles on the outer surfaces of SDs [44]. As seen in the SEM image of Figure 4D, no observable drug particles were found on the outer surface of the F2, which could be due to the homogeneous dispersion of ATO within the carrier’s matrix. Interestingly, we could also notice several small-sized SDs with small dents on the outer surface, which could have formed due to the rapid solvent evaporation during the spray-drying process. Based on these observations, it appears that the bulk drug was dispersed into the amorphous carriers in ATO-loaded SDs. Further, DSC and PXRD were employed to confirm the amorphous state of the ATO-loaded SD system.

**Figure 5:**
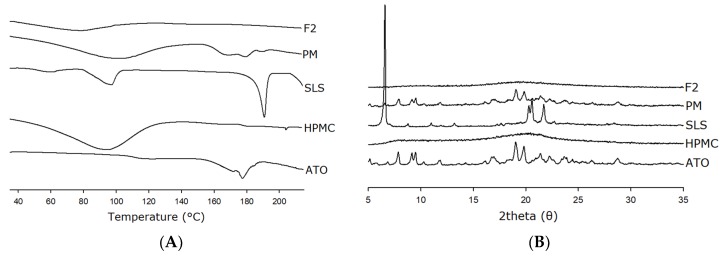
DSC (**A**) and XRD (**B**) curves of samples. PM represents a physical mixture of ATO, HPMC, and SLS at a 1:1:0.1 (*w*/*w*/*w*) ratio.

DSC was used to investigate the thermal behavior of free ATO, HPMC, SLS, and F2 (Figure 5A). Thermal analysis is a commonly used and useful tool, providing information regarding melting, phase transition, recrystallization, chemical degradation, and change in the specific heat capacity, and is useful for understanding the physiochemical characteristics of the dispersed drug inside the carriers [45]. Free ATO showed an endothermic peak at approximately 175 °C, corresponding to its melting point and indicating its crystalline nature [46]. Moreover, HPMC showed a broad endotherm at approximately 60–130 °C corresponding to its glass transition temperature (*T*_g_) [47], and SLS showed a sharp distinct endothermic peak at approximately 190 °C (melting) with a small broad peak at approximately 90 °C (loss of water) [48,49]. Further, PM showed an endotherm corresponding to the peaks of ATO, HPMC, and SLS, but with reduced intensity, indicating the presence of crystallinity and the absence of any strong interaction between drug and carriers. On the contrary, F2 did not produce any characteristic peaks in the DSC thermogram related to the bulk drug, which indicates the transformation of crystalline state ATO into its amorphous form in SD. Interestingly, a single broad endotherm peak with reduced intensity was observed for F2 at around 50–100 °C. This peak corresponds to the glass transition temperature (*T*_g_) observed due to the presence of HPMC in amorphous SD (Figure 5). However, the *T*_g_ value was broadened and shifted to a lower temperature range, presumably as a consequence of moisture loss from the F2 formulation. Similar peaks have been reported previously in studies using HPMC [47]. Generally, the presence of moisture lowers the temperature at which the transition occurs and also broadens the *T*_g_ range over which is it seen. These DSC results suggest a strong interaction between the drug and carriers due to molecular dispersion of drug into carrier matrix, which may have resulted in the amorphous nature of the ATO-loaded solid dispersion, and therefore in the absence of the drug characteristic melting peak.

Additionally, PXRD was conducted to further elucidate the amorphous nature of the SD. Figure 5B illustrates the PXRD patterns of free ATO, HPMC, SLS, PM, and F2. The diffraction pattern of free drug showed numerous sharp peaks, mostly at approximately 7°–30° of 2θ, suggesting highly crystalline characteristics of ATO [31]. HPMC and SLS showed numerous intrinsic peaks, and, in particular, SLS showed high-intensity peaks at approximately 5° and above 20°, due to its crystalline nature. Additionally, X-ray diffraction pattern of PM showed most of the major characteristic peaks as in those of the pure drug, HPMC, and SLS, suggesting its crystalline nature. In contrast, no characteristic diffraction peaks corresponding to free drug or carriers were present in F2. Thus, absence of any diffraction peaks in F2 is further evidence of the amorphous nature of ATO in the ATO-loaded SD, consistent with the DSC and SEM results.

Stable crystal lattice of drug molecules shows problems in solubilization due to its higher lattice energy. The reason for improved solubility and in vitro dissolution rate could be associated with the amorphous nature of ASDs (F2). In the amorphous form, molecules have the highest free energy and entropy resulting in superior molecular mobility; therefore, it requires less external energy for dissolution compared to its crystalline counterpart [16]. Therefore, changing the solid-state properties of drugs from crystalline to amorphous forms renders distinct advantage with regards to solubility.

### 3.5. Pharmacokinetic Studies

**Figure 6:**
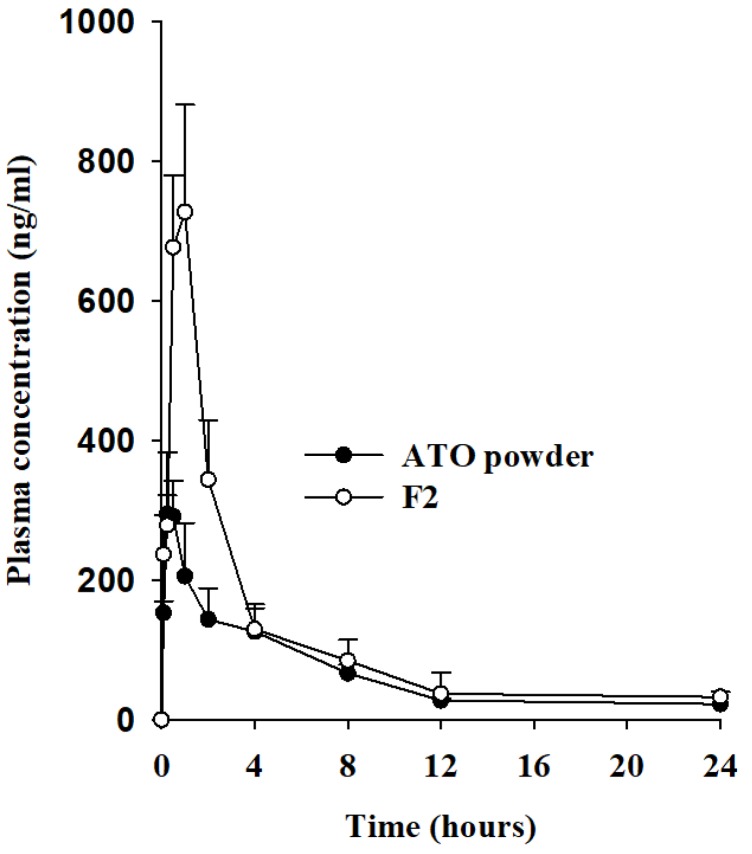
Plasma concentration–time profiles of ATO after oral administration of free drug or solid dispersions in rats. Each value represents the mean ± S.D. (*n* = 6). *p < 0.05 compared to free ATO.

The bioavailability of ATO in ternary ASD was assessed in rats. Figure 6 shows the mean plasma concentration versus time profiles of ATO after oral administration of free ATO or ATO-loaded SD (F2) at a dose of 30 mg/kg ATO. (cf. Figure S1 Supporting Information). The pharmacokinetic parameters are listed in Table 2. As shown in Figure 6, the total plasma concentrations of ATO were significantly higher in rats administered the F2 formulation than in those administered free drug powder alone, at all time points. The increase in bioavailability of a hydrophobic drug tends to be directly proportional to increase in overall bioavailability. The maximal ATO plasma concentration (*C*_max_) due to ATO-loaded SD (F2) (786.23 ± 84.72 ng/mL) was remarkably higher than that due to free ATO (328.83 ± 46.56 ng/mL) (Table 2). In addition, the AUC of free drug and the F2 formulation was 1557.34 ± 221.46 h·ng/mL and 2621.60 ± 318.99 h·ng/mL, respectively. Both the *C*_max_ and AUC of ATO-SD were approximately 2.4- and 1.68-fold higher compared to those of free ATO. However, there was no significant differences in the *T*_max_, *t*_1/2_, or *K*_el_ values of free drug and F2. These data show that use of a ternary ATO-loaded ASD results in substantial improvement of ATO absorption, possibly due to increased ATO solubility and dissolution rate in the gastrointestinal tract, and the amorphous nature of ATO in ASD. In clinical practice, in order to attain the same level of pharmacotherapeutic actions with ATO, the therapeutic dose of ATO in ATO-loaded SD could be now reduced by 1.68-fold. Hence, this could offer several benefits, including reduction in dose intake by patients (generally higher amount of API) and excipients associated with conventional oral formulations, fewer side effects than conventional dosages, and improved cost-effectiveness and overall patience compliance.

## 4. Conclusions

In the present study, hydrophilic polymer was successfully used to encapsulate the hydrophobic drug ATO into the solid dispersions via a spray-drying technique. We found increased solubility and improved dissolution profile of ATO in the prepared SD, due to efficient molecular dispersion of ATO in the hydrophilic carrier matrix and the conversion of the drug from a crystalline state to its higher energy amorphous form in the SD. The presence of only a single *T*_g_ observed over the entire DSC scan of F2 indicates good drug carrier miscibility in the prepared ASD. The pharmacokinetic parameters of the ATO-loaded SD were significantly higher than those of the free drug because of increased bioavailability, largely due to enhanced drug concentrations in the gastrointestinal (GI) tract. Hence, this could offer numerous benefits in a clinical setting, including the reduction in dose intake by patients (generally, a higher amount of API and excipients is associated with conventional oral formulations), resulting in fewer side effects and greater cost-effectiveness, and leading to improved patience compliance. Overall, our in vitro data, the results of the physical characterization of the SDs, along with supporting in vivo data show significant potential in the use of HPMC as a hydrophilic matrix-former for amorphous solid dispersion systems to enhance biopharmaceutical performance of poorly water-soluble drugs.

## Figures and Tables

**Table 1:** Composition of various solid dispersions prepared using various polymer-to-surfactant ratios.

Formulation	Drug (g)	HPMC (g)	SLS (g)
F1	1	0.5	0.1
F2	1	1	0.1
F3	1	2	0.1
F4	1	4	0.1
F5	1	8	0.1

**Table 2:** Pharmacokinetic parameters.

Formulations	ATO	F2
AUC (h·ng/mL)	1557.34 ± 221.46	2,621.60 ± 318.99 *
*C*_max_ (ng/mL)	328.83 ± 46.56	786.23 ± 84.72 *
*T*_max_ (h)	0.40 ± 0.14	0.70 ± 0.27
*t*_1/2_ (h)	4.15 ± 0.54	4.64 ± 1.45
*K*_el_ (h^−1^)	0.17 ± 0.02	0.17 ± 0.09

* *p* < 0.05 compared with free ATO. Each value represents the mean ± S.D. (*n* = 6).

## Supplementary Materials

The following are available online at https://www.mdpi.com/1999-4923/11/9/461/s1, Figure S1: Plasma concentration (log scale)–time profiles of ATO after oral administration of free drug or solid dispersions in rats.
