# Supplementary material for: Spray-Dried Amorphous Solid Dispersions of Atorvastatin Calcium for Improved Supersaturation and Oral Bioavailability
Source: Pharmaceutics. 2019 Sep 6;11(9):461. doi: 10.3390/pharmaceutics11090461 (PMC6781288; doi:10.3390/pharmaceutics11090461)
Supplement: Supplementary file 1 [file pharmaceutics-11-00461-s001.pdf]

pharmaceutics

Supplementary Materials: Spray-Dried Amorphous Solid Dispersions of Atorvastatin Calcium for Improved Supersaturation and Oral Bioavailability

Jaewook Kwon, Bhupendra Raj Giri, Eon Soo Song, Jinju Bae, Junseong Lee and Dong Wuk Kim

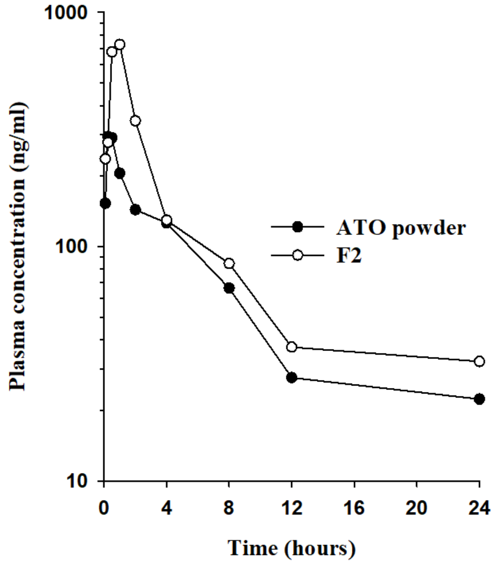

Figure S1. Plasma concentration (log scale)–time profiles of ATO after oral administration of free drug or solid dispersions in rats.
